# Supplementary material for: Concordance between the In Vivo Content of Neurospecific Proteins (BDNF, NSE, VILIP-1, S100B) in the Hippocampus and Blood in Patients with Epilepsy
Source: Int J Mol Sci. 2023 Dec 29;25(1):502. doi: 10.3390/ijms25010502 (PMC10779095; doi:10.3390/ijms25010502)
Supplement: Supplementary file 1 [file ijms-25-00502-s001.zip › Supplementary_.pdf]

Table S1. Patients' clinical information.

ID	*Age*	*Gender*	*Disease duration, years*	*Average seizure frequency per month*	*Average seizure duration, s*	*AEDs before surgery*	*Resection size*	*Histology*	*Engel (ILAE), scales*	*Biochemical analysis*	*IHC analysis*
1	56	F	42	5.5	N/A	1	a	HS	1A (class 1)	BDNF, NSE, S100B	NSE, S100B
2	23	F	13	11	60	2; 4	b	HG	1A (class 1)	BDNF, NSE, S100B	BDNF, NSE, S100B
3	26	M	23	1.5	45	2	b	HG	4C (class 6)	BDNF, NSE, S100B	BDNF, NSE, S100B
4	29	M	28	10	150	2	b	HG	1A (class 1)	BDNF, NSE, S100B	BDNF, NSE, S100B
5	34	M	9	4.5	45	5; 6	c	HS	1A (class 1)	BDNF, NSE, S100B	BDNF, NSE, S100B
6	32	F	7	14	35	2; 7	b	HG	1A (class 1)	BDNF, S100B	BDNF, NSE, S100B

7	28	F	28	1	315	2; 7	b	HG	3A (class 4)	BDNF, NSE, S100B	BDNF, NSE, S100B
8	24	M	24	7	450	4	b	HS	2B (class 3)	BDNF, NSE, S100B	BDNF, NSE, S100B
9	35	M	18	2	15	2; 5	b	HG	1B (class 2)	BDNF, NSE, S100B	BDNF, NSE, S100B
10	43	F	20	3.5	60	7; 8; 9	b	HG	1B (class 2)	BDNF, NSE, S100B	BDNF, NSE, S100B
11	24	M	17	3.5	240	2; 7	b	HS	1A (class 1)	BDNF, NSE, S100B	BDNF, NSE, S100B
12	34	F	11	7	5	7; 8	d	HG	4C (class 6)	BDNF, NSE, S100B	BDNF, NSE, S100B
13	27	F	16	5.5	80	2; 4	b	HG	1A (class 1)	BDNF, NSE, S100B	BDNF, NSE, S100B
14	33	F	22	5.5	150	5	c	HG	3A (class 4)	BDNF, NSE, S100B	BDNF, NSE, S100B
15	34	M	10	4	60	2; 4	b	HS	1A (class 1)	BDNF, NSE, S100B	BDNF, NSE, S100B

16	28	F	24	4.5	180	4; 5; 6; 7	b	HG	4B (class 5)	BDNF, NSE, S100B	BDNF, NSE, S100B
17	32	M	18	9	90	7; 10	b	HS	3A (class 4)	BDNF, NSE, S100B	BDNF, NSE, S100B
18	35	M	21	3.5	120	5; 6; 7	b	HG	1A (class 1)	BDNF, NSE, S100B	BDNF, NSE, S100B
19	28	F	5	90	90	2; 4	c	HS	1A (class 1)	BDNF, NSE, S100B	BDNF, NSE
20	36	M	11	6	70	7; 9; 10	b	HS	1A (class 1)	BDNF, NSE, S100B	BDNF, NSE
21	49	M	47	2	120	7; 9	b	HG	1A (class 1)	BDNF, NSE, S100B	BDNF, NSE
22	27	M	24	120	210	2; 5; 7; 9	b	HG	3A (class 4)	BDNF, NSE, S100B	BDNF, NSE
23	29	F	28	150	90	2; 4; 5; 9	b	HG	3A (class 4)	BDNF, NSE, S100B	BDNF, NSE
24	38	F	25	8	60	2; 4	b	HS	1B (class 2)	BDNF, NSE, S100B	BDNF, NSE

25	23	M	16	4	78	2; 4	b	HS	2B (class 3)	BDNF, NSE, S100B	BDNF, NSE
26	35	M	9	30	60	2; 4	b	HS	3A (class 4)	BDNF, NSE, S100B	BDNF, NSE
27	30	F	16	60	60	1; 2; 4; 5; 7; 8	b	HG	3A (class 4)	BDNF, NSE, S100B	BDNF, NSE
28	31	F	3	11	60	4; 8	b	HS	1A (class 1)	BDNF, NSE, S100B	BDNF, NSE
29	38	M	33	6	68	1; 2	b	HS	1A (class 1)	BDNF, NSE, S100B	BDNF, NSE
30	19	M	16	1.5	150	6; 9	b	HG	1A (class 1)	BDNF, NSE, S100B	BDNF, NSE
31	39	M	26	2.5	120	2; 8	b	HG	3A (class 4)	BDNF, NSE, S100B	BDNF, NSE
32	35	F	20	10	180	8; 9	b	HS	3A (class 4)	BDNF, NSE, S100B	BDNF, NSE, S100B
33	39	M	21	165	90	6; 7	b	HS	2B (class 3)	BDNF, NSE, S100B	BDNF, NSE, S100B

34	31	M	24	5.5	87	7; 9	b	HS	1A (class 1)	BDNF, NSE, S100B	BDNF, NSE
35	40	F	20	30	90	4	c	HS	1A (class 1)	BDNF, NSE, S100B	BDNF, NSE, S100B
36	37	F	28	3.5	193	4; 5	b	HS	4B (class 5)	BDNF, NSE, S100B	BDNF, NSE, S100B
37	31	F	19	12	75	2; 3; 4	b	HS	1B (class 2)	BDNF, NSE, S100B	BDNF, NSE, S100B
38	39	M	19	1.5	77	4; 5; 9	b	HS	4B (class 5)	BDNF, NSE, S100B	BDNF, NSE, S100B
39	41	F	33	210	90	1; 2; 4	b	HS	1A (class 1)	BDNF, NSE, S100B	BDNF, NSE, S100B
40	39	M	28	1.5	30	7; 9	b	HS	3A (class 4)	BDNF, NSE, S100B	BDNF, NSE, S100B
41	47	M	7	2	180	5; 7	b	HS	1A (class 1)	BDNF, NSE, S100B	BDNF, NSE, S100B
42	39	F	28	20	900	4	b	HS	4B (class 5)	-	BDNF, NSE, S100B

43	47	M	44	2	320	4; 5; 7	b	HS	3A (class 4)	-	BDNF, NSE, S100B
44	29	M	6	30	60	4	b	HS	1A (class 1)	-	BDNF, NSE, S100B

Abbrevations: M - male, F - female, AEDs - anti-epileptic drugs, HS - hippocampal sclerosis, HG - hippocampal gliosis, IHC analysis - immunohistochemical analysis, N/A - information is not available

Supplementary 1. Anti-epileptic drugs.

Number in Table S1	***Pharmacologic Class***	***Mechanism of Action***
1	Barbiturates and derivatives	Stimulate the inhibitory activity of GABAergic receptors
2	Carboxamide’s derivatives	Stimulate the inhibitory activity of GABAergic receptors; regulation of ion channels
3	Benzodiazepine’s derivatives	Stimulate the inhibitory activity of GABAergic receptors
4	Other anti-epileptic drugs / Levetiracetam	Regulation of ion channels

5	Other anti-epileptic drugs / Lamotrigine	Regulation of ion channels
6	Other anti-epileptic drugs / Perampanel	Reduce the excitatory activity of NMDA receptors
7	Valproic acid's derivatives	Stimulate the inhibitory activity of GABAergic receptors
8	Other anti-epileptic drugs / Topiramate	Stimulate the inhibitory activity of GABAergic receptors
9	Other anti-epileptic drugs / Lacosamide	Regulation of ion channels
10	Other anti-epileptic drugs / Zonisamide	Stimulate the inhibitory activity of GABAergic receptors; regulation of ion channels

Supplementary 2. Resection size.

Sign in Table S1	***Resected brain areas***
a	the hippocampus; areas of basal parts of the temporal lobe

b	the hippocampus; areas of basal parts of the temporal lobe up to 5 (4.5-6) cm from the pole (basal and medial parts subepically); the amygdala
c	the hippocampus; the amygdala
d	the amygdala-hippocampal complex of the temporal lobe

Supplementary 3. Classification of outcomes after surgical treatment of epilepsy.

Engel's scale (1993)

- ***Class I: Free of disabling seizures***

A. Completely seizure free since surgery

B. Non disabling simple partial seizures only since surgery

C. Some disabling seizures after surgery, but free of disabling seizures for at least 2 years

D. Generalized convulsions with AEDs withdrawal only

- ***Class II: Rare disabling seizures ("almost seizure-free")***

A. Initially free of disabling seizures but has rare seizures now

B. Rare disabling seizures since surgery

C. More than rare disabling seizures since surgery, but rare seizures for the last 2 years

D. Nocturnal seizures only

- ***Class III: Worthwhile improvement***

A. Worthwhile seizure reduction,

B. Prolonged seizure-free intervals amounting to greater than half the followed-up period, but not <2 years
• ***Class IV: No worthwhile improvement***<br>A: Significant seizure reduction<br>B: No appreciable change<br>C: Seizures worse

ILAE classification (2001)

Class 1: Completely seizure free; no auras
Class 2: Only auras; no other seizures
Class 3: One to three seizure days per year; ± auras
Class 4: Four seizure days per year to 50% reduction of baseline seizure days; ± auras
Class 5: Less than 50% reduction of baseline seizure days to 100% increase of baseline seizure days; ± auras
Class 6: More than 100% increase of baseline seizure days; ± auras
